# Psychological and Physical Distress in Italian People during COVID-19 Pandemic: One Year Later

**DOI:** 10.3390/ijerph182312525

**Published:** 2021-11-28

**Authors:** Alessandra Impellizzeri, Maddalena Dilucca, Francesca Squillace, Rosanna Guarnieri, Antonella Polimeni, Gabriella Galluccio

**Affiliations:** 1 Department of Oral and Maxillofacial Sciences, Sapienza University of Rome, 00185 Rome, Italy; squillace.francesca15@gmail.com (F.S.); rosanna.guarnieri@uniroma1.it (R.G.); antonella.polimeni@uniroma1.it (A.P.); gabriella.galluccio@uniroma1.it (G.G.); 2 Department of Physics, Sapienza University of Rome, 00185 Rome, Italy; maddalena.dilucca@gmail.com

**Keywords:** COVID-19, psychological distress, physical distress, Italian people, COVID-19 pandemic, SARS-CoV-2, questionnaire COVID-19

## Abstract

The aim of this study was to evaluate the major life changes that Italian people experienced after one year of the COVID-19 pandemic. We assessed the psychological and physical impact of COVID-19 within one year of the pandemic situation, and its possible correlation with the positive COVID-19 trend in the Italian region. We invited Italian people to complete a cross-sectional, online survey within a three-week period from 14 March to 4 April 2021. The survey collected data on the participants’ stress and physical levels, attitude, perceived control, norms, personal and professional backgrounds, and place of stay in the last year. We used Student’s *t*-test and the software package GRETL for Windows to assess the association between the study outcome variables and the explanatory variables (stress, attitude, perceived control, and norms). All participants who declared a level of physical stress in their answer suffered from psychological stress, but not vice versa. The result to be highlighted is that this level of stress was found more in women and in the age range of 21–45 years.

## 1. Introduction

Severe acute respiratory syndrome coronavirus 2 started in December 2019 in Wuhan, China (Hubei Province) [1]. Italy was one of the first hit and worst affected European countries since the end of February [2]. On 11 March 2020, the World Health Organization declared it a pandemic [3]. The name given to the new 2019 coronavirus is severe acute respiratory syndrome coronavirus-2 (SARS-CoV-2), a *β* coronavirus (CoV), a new strain of coronavirus that was not previously identified in humans. The CoVs, which commonly infect mammals [4,5,6], are RNA viruses suggested to have pandemic potential due, in part, to their large genome and mutation rate [7]. The dominant mode of person-to-person transmission of SARS-CoV-2 is directly from the respiratory tract via droplets, or indirectly through contaminated objects or surfaces [1,8], which is important in clinical dentistry because many dental procedures produce aerosols and droplets that are contaminated with bacteria, viruses, and blood, and have the potential to spread infections to dental personnel and other people in the dental office [9,10,11]. This epidemic has had more than 3,223,142 cases and more than 102,145 deaths [12]. Thus, to control COVID-19 infection, the Centers for Disease Control and Prevention (CDC), the American Dental Association (ADA), and the World Health Organization (WHO) recommended practice guidelines for dental clinicians [13,14,15,16]. The virus has affected not only clinical practice, but all life aspects. The highly contagious nature of the epidemic and its awful outcomes led to lifestyle changes for everyone [17]. This new lifestyle included social distancing, avoiding public places, thorough and regular hand washing, and the mandatory wearing of face masks in public [18]. Adding to the fear of contracting the virus are the significant changes associated with new stress-inducing factors such as working from home, temporary unemployment, home-schooling of children, and lack of physical contact with other family members, friends, and colleagues [19]. Undoubtedly, these were stressful times, particularly since the stressor was new, and it was unknown how the pandemic would affect our future lifestyles and if/when we can resume our regular lives. This pervasive uncertainty makes it difficult to plan for the future and thus generates additional psychosocial stress [20]. Severe anxiety is associated with physical symptoms such as muscle tightening, hyperventilation, increased heart rate, sweating, trembling, fatigue, troubled sleeping, and gastrointestinal disorders in addition to impaired cognitive skills [21]; furthermore, the persistent, severe anxiety may affect physical and mental well-being [22,23]. The aim of this study was to advocate for an increased focus on mental health during the coronavirus pandemic by evaluating the most significant life changes on Italian people one year after the COVID-19 pandemic. We assessed the psychological and physical impact of COVID-19 within one year of the pandemic situation, and the possible correlation with the positive COVID-19 trend across the Italian region.

## 2. Materials and Methods

Recruitment of the Sample

We invited Italian people to complete a cross-sectional, online survey for 3 weeks, from 13 March 2021 to 4 April 2021. The survey collected data on the participants’ stress and physical activity levels, attitude, perceived control, norms, personal and professional backgrounds, and place of stay in the last year from 11 March 2020 to 2021. The questionnaire was developed and forwarded to Italian people in the Italian language and translated to English for the presentation of this research. The scientific bases for the development of the questions about the psychological and physical impact of COVID-19 on the Italian people were derived by consulting the scientific literature available on this subject. 

The questionnaire was uploaded online to the free survey platform Google Docs (web-based Google Docs Editors suite offered by Google, docs.google.com (accessed on 18 August 2021)) through a specially created user profile. 

The sample for the online questionnaire was chosen randomly. Each person had to enter with their personal email to participate in the survey to prevent the same individual from repeating the questionnaire several times. By setting these rules, we controlled the duplicity of responses from the same person.

The generated link was shared across different groups and the contact networks were shared on the main social channels (Instagram, Facebook, and WhatsApp), inviting Italian people to share the link with other persons and groups to widen the spread of the survey as much as possible. 

Variables and instruments

The inclusion criteria were people who spent the lockdown in Italy, and people between 18 and 70 years old; the exclusion criteria were all people who did not spend the lockdown in Italy, and people outside of the 18–70 year age range.

The responses were validated only for fully completed questionnaires; the system automatically rejected incomplete questionnaires. Participants were asked to select only one response per question, and they were allowed to make one submission. To ensure confidentiality, no IP addresses or emails were collected. Preceding the questionnaire was a brief introduction explaining the purpose of the study, assuring participants of the confidentiality of their responses, and emphasizing that their participation was voluntary. Afterward, the survey was tested for face and content validity by four academics who were not involved in the study to ensure the clarity and relevance of the questionnaire. The data collected were absolutely anonymous, and tracing the identity of the subjects was not possible. 

Procedure and description of the study

For this study, a total of 30 open- and close-ended questions were developed (Table S1). 

Five questions helped to obtain a profile of the participants (sex; age group; type of clinical activity, private; territorial provenance, specifying the name of their region). Six questions were intended to evaluate their perception of the presence or absence of infected cases in their region, if there were changes in the region of residence, effective management, and respect of special measures implemented in their region to prevent pandemic diffusion.

Seven questions helped to obtain a psychological profile of the participants with questions on the future perspectives, smart-working frequency, personal level of psychological and physical distress, individual or family depression history, desire to undertake social relationships, and the necessity of medical psychological support. Four questions were about COVID-19 infection and vaccine inoculation. Another component of the study was assessed using the last eight questions, which were directed toward understanding the influence of the coronavirus epidemic on the participants’ dental clinical activity: appearing to be worried or not to be worried about possible infections with coronavirus during dental procedures, effective appointment numbers since the coronavirus outbreak onset, adoption of special measures taken during professional activity since the coronavirus emergency started in Italy, and what prevention methods were used. 

## 3. Analysis

Differences between the true responses of the participants were allocated to the questionnaire and a randomized version (random model) of responses were calculated to test the validity of the questionnaire. Specifically, we tested the level of significance using Student’s *t*-test, considering a *p*-value < 0.05 (the statistical significance level was set at 5 percent). The software package GRETL for Windows was used for statistical analysis [24].

## 4. Results

### General Information

Overall, 2075 participants completed the online survey. Of these, 523 (25%) were men and 1552 (75%) were women. Geographically, 317 (15.3%) of the responders were from North Italy, 1027 (49.5%) lived in the center of Italy, and 731 of the responders were located in South Italy or the Islands (35.2%). About half (60.2%) of responders were 21–45 years old and about 40% were over 45 years of age. These details are reported in Table 1.

## 5. Perception of the COVID Pandemic

We investigated the correlation between perceived psychological impact and the increase in the number of COVID-19 cases. To analyze this situation, we focused our attention on each of the questions in the questionnaire. First, the questions were related to the general information of the sample. In question 6, all participants responded that there were COVID cases in their region as confirmed by Civil Protection data. There were no regions in Italy without any cases, thus the questionnaire data is consistent. In question 7, when asked if someone has moved to another region since the COVID-19 pandemic began, more than half of the participants replied that they had made a change. If the answer to question 7 was yes, in question 8, the participants stated the region to which they moved. These data are not correlated with the region of origin or with the number of cases of departure in the region. It is important to remember that in Italy, the average migratory flow of the population and, in particular, of young people, is not equally distributed in all areas of the nation. The average flow of people moving from the south to the north of Italy is, on average, greater than from north to south.

In question 9, people living in South Italy and the Islands reported having the perception of few infections in their areas, as they were less affected. In North Italy, the number of infections was very high, especially in Lombardy. Here, the perception of the pandemic was negative. 

**Figure 1:**
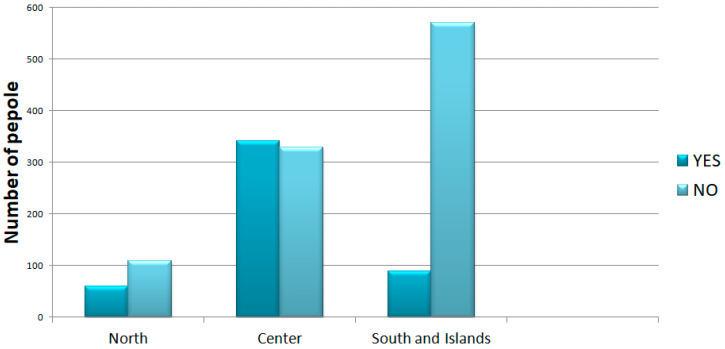
Question 10 asked if, compared to the other regions, participants thought that their region managed the spread of the pandemic better.

**Figure 2:**
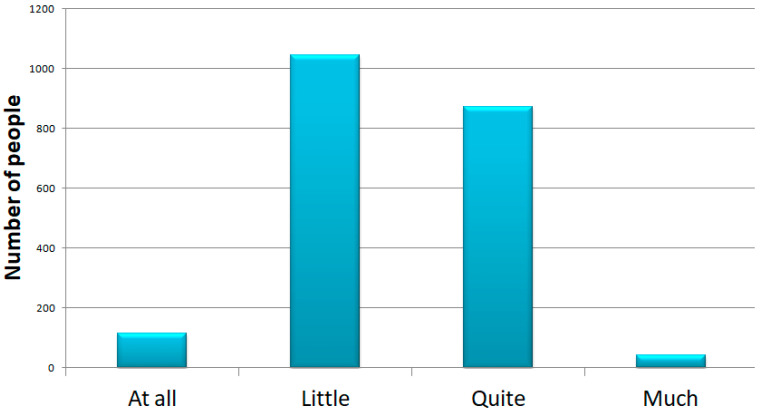
Question 11 asked if the anti-contagion regulations were respected in the respondent’s city.

In question 10, participants were asked if they thought that their region managed the spread of the pandemic better than the other regions. The perception of the pandemic was also disastrous in central Italy (see Figure 1). The perception factor could be commonly correlated to an economic factor and to the availability of health facilities, which allowed some regions of Italy to have fewer victims and cases of hospitalization. For what concerns question 11, we were interested in investigating the participants’ compliance with the rules during the period of the pandemic. These data are not correlated with the number of infections in the different areas, or with the number of inhabitants (Figure 2). 

## 6. Psychological Impact

To question 12, 475 of the participants replied that they will return to normal life after the pandemic within one year, 896 replied that this will happen within two years, 258 replied that we will not return to normal life, and 464 said they had no idea. When we analyzed these data, we considered the fact that most of the people who responded lived in North Italy, where the number of infections was greater. These data are correlated to the participants’ sector of work. As some work areas are more interesting than others, work cannot be considered among the main factors that caused any psychological stress.

**Figure 3:**
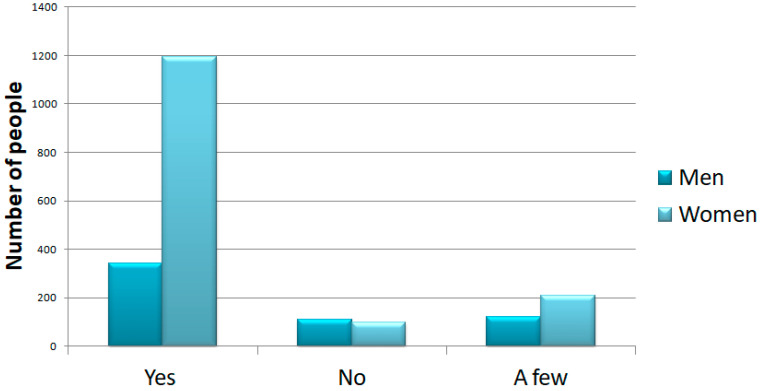
Question 14: increased level of psychological stress. In the top panel, we show the number of people that selected each response subdivided into men and women.

**Figure 4:**
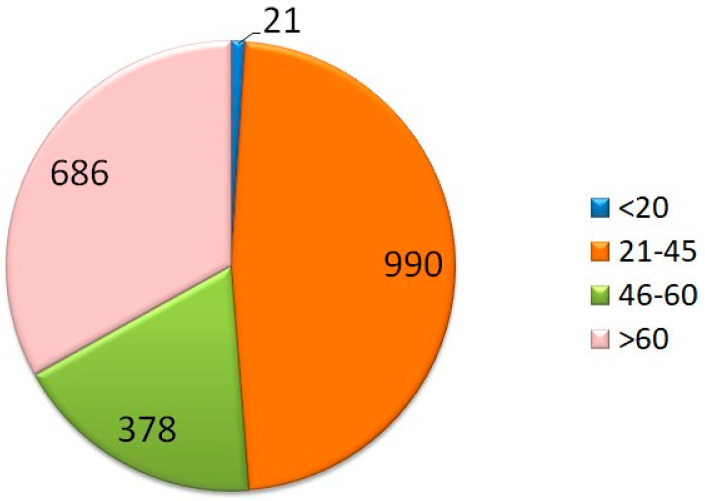
Question 14: increased level of psychological stress. In the bottom graph, we can see that the majority of participants that answered “yes”, were people aged 21 to 45 years old.

Questions 14 and 15 focused on physical and psychological stress. The results showed that 1537 (74%) of the participants answered that they felt they were in a situation of psychological stress, 208 (10%) answered no, and the other 330 responded saying low amounts of stress (16%). We showed that the majority of people suffered from psychological distress and that 70% of these were women; although it is important to note that the initial sample is composed by more women than men. Furthermore, we can see that the majority of people that answered “yes” were people ranging from 21 to 45 years of age (Figure 3 and Figure 4).

**Figure 5:**
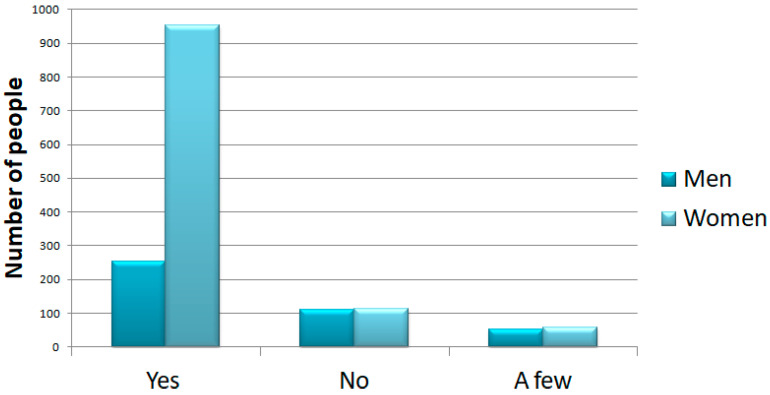
Question 14: increased level of physical stress. In this graph, we show the number of people that selected each response subdivided into men and women. Women reported a high level of physical stress.

On the other hand, 1206 (78%) of participants answered that they felt they were in a situation of physical stress. The results showed that 221 (15%) answered “no”, and the other 110 “a little” (7%). Again, women were the ones who reported the greatest physical stress (Figure 5).

An important result is that all people who declared a level of physical stress also stated that they suffered from psychological stress, but not vice versa. These data for stress are not correlated with the areas of living.

Question 16 is more interesting because it points out moments of depression that someone or someone in the participants’ household experienced during the pandemic. The responses showed that 902 (75%) of participants answered that they experienced moments of depression, 205 (17%) stated no, and 99 (8%) abstained from responding.

Question 17 asked the participants if they perceived that there was decreased desire to engage in social relationships; the answers were evenly distributed and do not suggest a prevalence of abstention or social propensities. In reference to this, it was asked if they had contacted a specialist for psychological support. The responses showed that 128 (10%) of participants answered that they required the care of a psychologist, 995 (83%) answered no, 52 (4%) said sometimes, and only 31 (3%) said that they were still being treated by a psychologist. Finally, they were asked if they had attended a vaccination clinic and if they had received the second dose of the vaccine: 552 replies yes, most of the other participants abstained from responding.

From a psychosocial point of view, while social connectedness decreased during the lockdown, probably because of isolation and social distancing, the virtual social community seemed to increase in the same period [24,25]. Our participants declared that they continued to go to work in person and few used smart working. Work and the large use of computers for smart working cannot be considered the factors that caused any depression or psycho-somatic stress. Previous studies already highlighted the impact of COVID-19 on general well-being and mental health [26]; in line with the literature that is currently available on the impact of COVID-19, we found that most of the people who participated in the questionnaire experienced a strong psychological and physical stress component. 

The latest data (https://www.epicentro.iss.it/mentale/epidemiologia-Italia (accessed on 18 August 2021)) instead indicate that in Italy, only about 6% of adults aged 18–69 years reported depressive symptoms. An important result is that all people who declared a level of physical stress answered that they suffered from psychological stress, but not vice versa. The result to be highlighted is that this level of stress was found to be higher women and in the 21–45 years age group. We know from the literature that female sex is associated with higher levels of psychological distress, including problems of depression, stress, and anxiety levels [27].

## 7. Conclusions

This is one of the first studies, to the best of our knowledge, that prospectively examined changes in psychological distress among a representative sample of adults as the COVID-19 crisis evolved in Italy. The WHO declared COVID-19 as a global pandemic on 11 March 2020, after the first coronavirus cases in Wuhan and as the viral disease swept into at least 114 countries and killed more than 4000 people. This emergency had a significant impact on socio-economic and psychological dimensions, in addition to the growing number of infected individuals and deaths. In Italy, the measures that were adopted to contain the diffusion of COVID-19 had a strong impact on people’s quality of life and mental health. The objective of this analysis was to quantify the psychological impact of this period on the general Italian population after one year into the lockdown. The study (2075 adults) was conducted for three weeks from 14 March to 4 April 2021. During the period of the lockdown, there was increased arousal mainly for negative emotions, but also for positive emotions, and the quality of life seemed to be reduced. 

We did not find a relevant correlation between the level of stress, the kind of job, or the area of living. This implies that the stress level of the individual during the pandemic was independent of the surrounding or social situations and the environment in which the individual found themselves and lived. Furthermore, participants did not believe that they would soon return to everyday life without the anti-COVID norms. They also declared that they had experienced periods of depression themselves or within their family members, but despite this, they did not seek psychologic treatment. These data do not agree with the data reported in the literature, according to which there was a significant increase in the number of requests or help phone lines and psychological support in France, Italy, and Spain. Despite several limitations due to the small number of people who participated in the survey and the individual emotional variability at the moment of completing the survey, our study provides information about the immediate psychological responses of Italy’s general population to the COVID-19 pandemic. Our results help gauge the psychological burden on the community to help minimize the impact.

## Figures and Tables

**Table 1:** Demographic characteristics of the sample.

		N	%
Sex	Male	523	25
	Female	1552	75
Regions of Italy	North	317	15.3
	Center	1027	49.5
	South and Islands	731	35.2
Age (years)	<20	52	2.5
	21–45	1210	60.3
	46–60	527	26.3
	>60	220	10.9
Work	Freelancer Employee/HousewifeOther	468	22.5
		953	45.9
		149	7.1
		506	24.3

## Data Availability

Not applicable.

## Supplementary Materials

The following are available online at https://www.mdpi.com/article/10.3390/ijerph182312525/s1, Table S1. Questionnaire attached in a supplementary file.
