# Supplementary material for: Psychological and Physical Distress in Italian People during COVID-19 Pandemic: One Year Later
Source: Int J Environ Res Public Health. 2021 Nov 28;18(23):12525. doi: 10.3390/ijerph182312525 (PMC8656865; doi:10.3390/ijerph182312525)
Supplement: Supplementary file 1 [file ijerph-18-12525-s001.zip › ijerph-1439271-supplementary.pdf]

Table S1. Questionnaire attached in a supplementary file.

1. Are you a man or a woman? *

A) Man
B) Woman

2. What age group do you belong to? *

A) <20 years
B) between 21 and 45 years old
C) between 46 and 60 years old
D)> 60 years

3. What kind of work do you do? *

A) Freelance professional
B) Employee
C) Unemployed
D) Other

4. In what part of Italy are you resident / domiciled? *

A) Northern Italy
B) Central Italy
C) Southern Italy & Islands

5. Specify the name of your region of residence / domicile below: *

—————-

6. Have there been any cases of COVID-19 in your region? *

A) Yes
B) No
C) I don't know

7. Since the COVID-19 pandemic started, have you moved to another region? *

A) Yes
B) No

8. If the answer to the previous question is positive, the region you moved to belongs to:

A) Northern Italy
B) Central Italy
C) Southern Italy & Islands

9. What is your perception of COVID-19 infections in your region? *

A) no contagion
B) few infections
C) many infections

10. Do you think that, compared to other regions, the one where you are managed the spread of the Pandemic better? *

A) Yes
B) No
C) I don't know

11. In your perception, are anti-contagion policies respected in your city? *

A) Not at all
B) A little
C) Enough
D) A lot

12. How soon do you think we can return to normal, pre-pandemic life? *

A) Within a year
B) Within two years
C) We will never go back to normal
D) I have no idea

13. Since the spread of COVID-19, have you decreased working from the office, in favour of Working from Home (WFH)? *

A) Yes
B) Yes, only after the increase in the incidence of cases in my region
C) No, my work does not include the possibility of WFH
D) No, but I would have loved to
E) I am unemployed

14. Do you think that in the last year, your level of psychological stress has increased? *

A) Yes
B) No
C) A little

15. Do you think that in the last year, your level of physical stress has increased? *

A) Yes
B) No
C) A little

16. Do you think you or someone in your family have experienced moments of depression related to the pandemic situation of the last year? *

A) Yes
B) No
C) I don't know

17. Compared to a year ago, before the beginning of the pandemic, do you think your desire to engage in social events has decreased? *

A) Not at all
B) A little
C) Enough
D) A lot

18. In the last year, did you need to go to a specialist for psychological support? *

A) Yes
B) No
C) Sometimes
D) I continue to go, even today

19. Have you had COVID-19? *

A) Yes
B) No

20. Has anyone in your family been infected with COVID-19? *

A) Yes
B) No

21. Were you part of the first vaccination campaign, and have you already got the second dose of the vaccine? *

A) Yes
B) No
C) I refused to be vaccinated

22. If the answer to the previous question is yes, do you feel psychologically safer, in case of a hypothetical contagion from COVID-19?

A) Yes
B) No
C) I don't know

23. Did you need to go to the dentist during this pandemic year? *

A) Yes
B) No
C) I went only for Indifferent Urgencies

24. For treatments, you have gone to dental facilities:

A) Public (hospitals, local health authorities, affiliated centers)
B) Private Clinics

25. How many times have you been to the dentist in the last year of the pandemic? *

A) 0
B) between 1-3 times
C) more than 3 times

26. Do you think a dental visit is more dangerous for the possibility of transmission of COVID-19 than any other specialist medical visits? *

A) Yes, absolutely
B) No, the risk is lower
C) No, it is as dangerous as any other specialist medical visits
D) I don't know

27. Are you concerned about the possibility of receiving dental visits / treatments safely?

A) Yes
B) No

28. Since the spread of the COVID-19 in our country, has your dentist taken precautions or taken special measures while your professional activity? *

A) Yes
B) No

29. Will you continue to go to the dentist with confidence in the coming months? *

A) Yes
B) No

30. If you gave up going to the dentist, did you do it for fear of getting infected?

A) Yes
B) No
